# How Well Do Young Male Semi-Professional Soccer Players Sleep During an Afternoon Nap?

**DOI:** 10.3390/clockssleep8030044

**Published:** 2026-07-15

**Authors:** Charli Sargent, Georgia Romyn, Michele Lastella, Dean J. Miller, Gregory D. Roach

**Affiliations:** 1 Appleton Institute for Behavioural Science, Central Queensland University, P.O. Box 42, Wayville, SA 5034, Australia; georgiaromyn@gmail.com (G.R.); m.lastella@cqu.edu.au (M.L.); d.j.miller@cqu.edu.au (D.J.M.); greg.roach@cqu.edu.au (G.D.R.); 2 S.P.O.R.T. Research Cluster, Rockhampton Campus North, Central Queensland University, Bruce Highway, North Rockhampton, QLD 4701, Australia

**Keywords:** daytime sleepiness, sport, polysomnography, slow-wave sleep, REM sleep, sleep latency, football

## Abstract

Athletes often experience situations in which they do not obtain adequate sleep at night. One strategy to overcome this issue is to supplement nighttime sleep with a daytime nap. However, little is known about how well athletes sleep during daytime naps. The aims of this study were to (i) determine the ability of athletes to fall asleep during an afternoon nap and (ii) examine the composition of the sleep obtained. In a counterbalanced, repeated-measures cross-over design, 12 young male semi-professional soccer players were given a 1 h (15:00–16:00) or 2 h (14:00–16:00) nap opportunity following a normal night’s sleep (7–8 h time in bed) in a laboratory. Sleep was monitored using polysomnography and variables were compared between naps using generalized estimating equation models. In the 1 h nap, participants took 6.5 min to fall asleep and spent 52/60 min asleep (87% efficiency); in the 2 h nap, participants took 7.5 min to fall asleep and spent 105/120 min asleep (87% efficiency). There was no difference in wake after sleep onset (*p* = 0.168) or time spent in stage N3 sleep (*p* = 0.110) between the 1 h and 2 h nap, but participants fell asleep faster (*p* < 0.001), obtained more sleep (*p* < 0.001), and spent more time in stage N1 sleep (*p* = 0.003), stage N2 sleep (*p* < 0.001), and Stage REM sleep (*p* = 0.004) in the 2 h nap. Athletes sleep well during 1 h and 2 h afternoon naps and could use this as a strategy to help meet sleep duration recommendations (i.e., 7–9 h).

## 1. Introduction

Training and competition form a major part of the life of an elite athlete. However, in some situations, the time of day that training and/or competition occurs can interfere with the time available for sleep at night. For example, on the nights prior to early morning training sessions, athletes spend 19% less time in bed and obtain 27% less sleep compared to days off [1] and on nights immediately after evening competition, athletes spend 37% less time in bed and obtain 36% less sleep compared to nights after daytime competition [2]. One of the many challenges faced by elite athletes is how to deal with the loss of sleep on the day(s) following early morning training sessions and/or evening competition [2,3,4].

Napping is a strategy that is commonly used by individuals who do not obtain sufficient sleep. For example, individuals who regularly undertake shiftwork report napping as a major strategy for supplementing insufficient sleep [5,6]. Elite athletes frequently experience short sleep durations, but the prevalence of napping is low—most athletes (~43%) only nap once per week during a normal phase of training [1,7]. Additionally, some athletes report that it is difficult to initiate sleep during the day and are unsure whether they can obtain good quality sleep [8].

The impact of a daytime nap on subsequent physical performance in athletes has been examined in several studies [9,10,11,12,13,14,15,16,17,18,19,20]. However, in almost all of these, the amount and/or quality of sleep obtained in the nap is not assessed or reported [10,11,12,13,14,16,19]. Furthermore, the duration of the nap tends to be short (i.e., 20–40 min) [9,11,14,15,16,17], sleep is often restricted the night before the nap [11,12,14,15,16,19], and as part of the experimental design, the nap is either terminated once sleep onset occurs [17] or purposely disrupted if deep sleep occurs [15]. These approaches, while suitable for addressing a particular experimental aim, limit the ability to interpret the composition of sleep during daytime naps in athletes.

The composition of sleep during early-morning naps has been examined in six well-trained adults [20]. In a repeated-measures, cross-over design, the participants in this study were given a 90 min nap between 10:30–12:00 and 11:30–13:00 after an 80 min endurance training session at an intensity corresponding to 80% of predicted maximal heart rate. The primary aim of the study was to examine how well the participants slept following a morning endurance training session, and to determine whether the timing of the nap (10:30 vs. 11:30) had an impact on the content of the sleep. In both nap conditions, the participants fell asleep quickly (~8 min), converted ~75% of the nap opportunity to sleep, and obtained a similar amount of rapid eye movement sleep (~7 min); although the participants obtained twice as much stage 3 sleep in the nap at 11:30 (~14 min) than in the nap at 10:30 (~7 min). Early-morning naps present athletes with one option when attempting to supplement inadequate nighttime sleep, and although the participants in this study were able to initiate sleep quickly, sleep efficiency was low (~75%). The propensity for sleep is usually low in the morning because the homeostatic drive for sleep has dissipated during the previous night’s sleep and the circadian system is promoting alertness [21,22]. An alternative strategy for athletes, that has yet to be examined, is to schedule naps in the afternoon, when the propensity for sleep is higher than it is during the morning [23].

In a recent study, we demonstrated that it was possible to split an athlete’s sleep opportunity into a nighttime sleep (either 7 h or 8 h time in bed at night) and an afternoon nap (either 1 h or 2 h in duration starting at 14:00) with no negative consequences for total sleep time when compared to a standard sleep schedule (i.e., 9 h time in bed at night with no afternoon nap) [24]. In that study however, the afternoon naps were analyzed in combination with nighttime sleep opportunities. Here we report the characteristics of the 1 h and 2 h daytime naps, including the time taken to initiate sleep and the composition of sleep during the naps.

## 2. Results

On the night prior to the 1 h nap, participants were given an 8 h sleep opportunity and on the night prior to the 2 h nap, participants were given a 7 h sleep opportunity. Participants obtained more sleep on the night prior to the 1 h nap (7.3 ± 0.4 h) compared to the 2 h nap (6.3 ± 0.7 h; *z* = −3.059, *p* = 0.002), but obtained a similar amount of stage 3 sleep (8 h: 36.3 ± 8.6 min; 7 h: 30.4 ± 12.6; *t* = −0.569, *p* = 0.581) and REM sleep (8 h: 76.8 ± 31.5 min; 7 h: 91.3 ± 24.7 min; *t* = −1.659, *p* = 0.125). The sleep architecture observed for sleep episodes prior to both nap conditions was comparable to that of healthy male adults of a similar age [25].

There was no difference in REM onset latency between the 1 h and 2 h naps; however, sleep onset latency and stage N3 onset latency were significantly shorter in the 1 h nap than in the 2 h nap (Table 1 and Table 2). Participants obtained more sleep, and had better sleep efficiency, in the 2 h nap compared to the 1 h nap. There was no difference in wake after sleep onset between the two nap conditions (Table 1 and Table 2).

In absolute terms, participants obtained more stage N1 sleep, stage N2 sleep, and REM sleep in the 2 h nap compared to the 1 h nap but obtained a similar amount of stage N3 sleep (Table 1 and Table 2). When expressed as a percentage of total sleep time, the percentage of stage N1 sleep was not different between the 1 h and 2 h naps, but the percentages of stage N2 sleep, stage N3 sleep, and stage REM sleep were higher in the 2 h nap compared to the 1 h nap (Table 1 and Table 2). Participants experienced more arousals per hour of sleep, had more stage shifts and more awakenings in the 2 h nap compared to the 1 h nap, but there was no difference in subjective sleep quality between the two nap conditions (Table 1 and Table 2).

**Figure 1:**
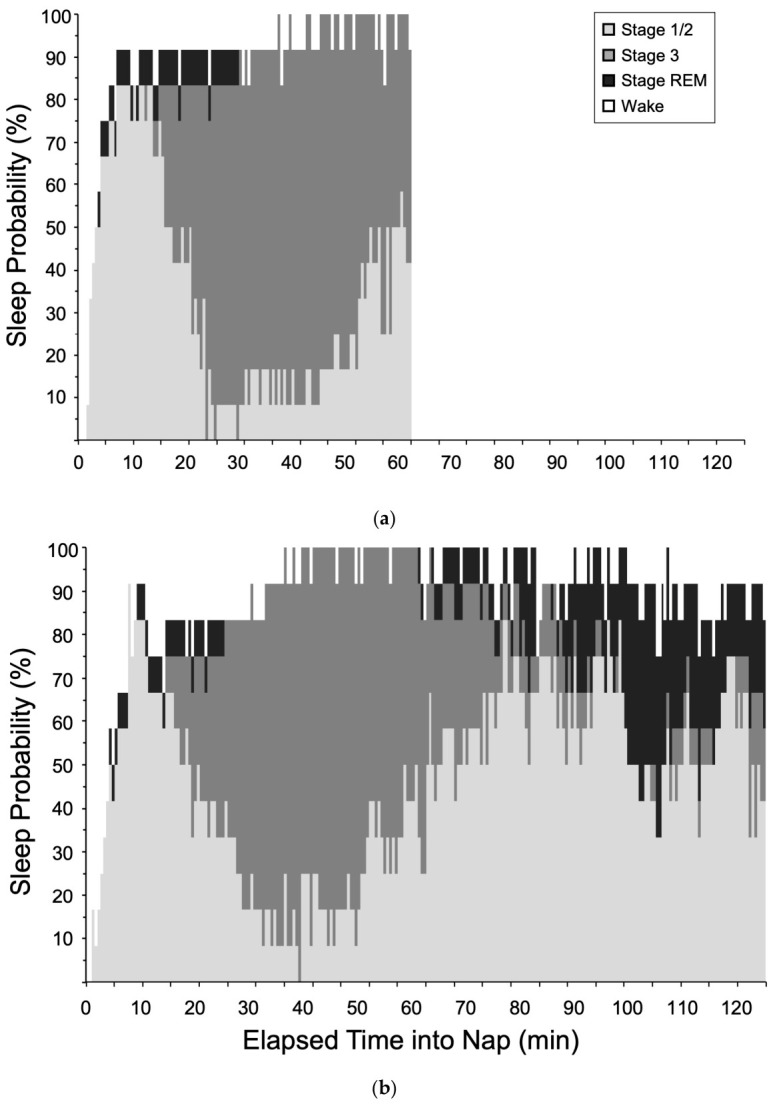
Sleep histograms representing the probability distribution of sleep stages in the (**a**) 1 h and (**b**) 2 h nap conditions. Data represent the percentage of epochs scored as Stage N1 and Stage N2 sleep (light grey bars), Stage N3 sleep (grey bars) and Stage REM sleep (dark grey bars) in 30 s epochs.

The probability distributions of sleep stages and wake were calculated for the 1 h and 2 h nap conditions (Figure 1). In both nap conditions, wake is predominant at the start of the nap, and then quickly declines. Stage N3 is predominant in the first hour of both naps and in the 2 h nap, stage N2 is predominant in the second hour of the nap. Some REM sleep occurs in the early part of the 1 h nap; in the 2 h nap, most of the REM sleep occurs in the last hour of the nap.

## 3. Discussion

Athletes often encounter situations that prevent them from spending adequate time in bed at night to obtain sufficient sleep [2,27,28,29]. When this occurs, it may be possible to supplement nighttime sleep with a daytime nap. Daytime napping is commonly used by populations that are vulnerable to sleep loss (e.g., shiftworkers), but few athletes nap on a regular basis [1,7]. The aim of the present study was to examine the ability of athletes to fall asleep during a 1 h or 2 h afternoon nap following a normal night’s sleep (i.e., 7–8 h time in bed) and to examine the composition of sleep during the naps.

In both the 1 h and 2 h naps, sleep was initiated quickly and the athletes were able to convert most of the nap opportunity to sleep. On average, the athletes took ~7 min to fall asleep (range: 2–41 min) and spent 52/60 min asleep in the 1 h nap; and took ~8 min (range: 1–39 min) and spent 105/120 min asleep in the 2 h nap. Similar sleep latencies have been reported for slightly older sub-elite athletes when given a 20 min nap in the early afternoon (i.e., 13:30) after obtaining ~6.6 h of sleep the night before [17] and slightly older physically fit male volunteers when given a 90 min nap in the morning (i.e., 10:30–11:30) after ~6.8 h of sleep the night before [20]. Taken together, these results indicate that athletes can initiate sleep quickly during a daytime nap—regardless of whether it occurs in the morning or in the afternoon.

The composition of sleep during daytime naps in athletes has been examined in very few studies. Davies et al. [20] described the composition of sleep during early-morning naps in six well-trained male adults. In a repeated-measures, cross-over design, the participants were given a 90 min nap between 10:30–12:00 and 11:30–13:00 after an 80 min endurance training session (80% of predicted maximum heart rate). The participants obtained a similar amount of REM sleep (~7 min) in both nap conditions but obtained twice as much slow wave sleep in the nap at 11:30 (~14 min) compared to the nap at 10:30 (~7 min). The amount of time in bed that was converted to sleep during the nap (i.e., sleep efficiency) was similar (albeit moderate) between the two nap conditions (~75%). In comparison, sleep efficiency in the present study was much higher in both the 1 h and 2 h naps (~87%), the participants obtained more than twice as much slow wave sleep in both naps (~33 min), and the participants obtained ~13 min of REM sleep in the 2 h nap. The differences between these two studies most likely reflect differences in the timing and duration of the naps. Specifically (i) slow wave sleep increases as a function of time awake [14], and thus should be higher in an afternoon nap (i.e., after 6–7 h of wakefulness) compared to a morning nap (i.e., after 4–5 h of wakefulness); (ii) healthy individuals cycle between non-REM sleep and REM sleep every 90 min [30], and thus a 2 h nap should contain more REM sleep than a 90 min or a 1 h nap; and (iii) sleep propensity is higher in the afternoon than in the morning, and thus sleep efficiency should be higher in an afternoon nap compared to a morning nap [22,23].

In the present study, the composition of sleep was somewhat similar between the nap conditions. However, the proportion of slow wave sleep was higher in the 1 h nap than in the 2 h nap (+20%) and more REM sleep occurred in the 2 h nap than in the 1 h nap (+8 min). Given the role of different sleep stages for recovery and performance, daytime naps could be timed and structured to suit a particular purpose. For example, slow wave sleep is thought to play a role in the recovery from exercise [31]. A 1 h nap in the afternoon could be used to facilitate recovery when training and/or competition loads are high. In contrast, REM sleep is associated with improved learning and memory [32]. A 2 h nap in the afternoon could be used to facilitate learning when athletes are required to develop new skills and/or consolidate new information. However, markers of recovery and aspects of learning and memory were not assessed after naps in this study. In the future, it will be interesting to determine whether daytime napping—and the associated composition of sleep during the nap—could be used strategically to improve aspects of performance and/or recovery in athletes.

One point of consideration for athletes who wish to implement daytime napping is the effect it may have on the subsequent night’s sleep. Sleep is regulated by two processes—a circadian process generated by an endogenous pacemaker, and a homeostatic process that reflects the pressure for sleep that builds up during sustained wakefulness and dissipates during sleep periods [21]. If a nap is taken during the day, the pressure for sleep is partially reduced [33]. For some individuals, this reduction in sleep pressure may increase sleep latency later that night and/or reduce the amount of non-REM sleep obtained, even if bedtime occurs several hours after the nap [34]. In the present study, the 1 h and 2 h naps were administered in a counterbalanced order, so it was not possible to assess the impact of naps on subsequent nighttime sleep. In situations where an athlete is using a daytime nap to supplement inadequate nighttime sleep (i.e., <8 h), there may be little or no impact of the nap on nighttime sleep. However, if an athlete is using daytime napping as a strategy to increase total sleep duration beyond the usual recommendation (i.e., >8 h), the duration and/or quality of the subsequent nighttime sleep may be compromised. This is an important issue because athletes are often ‘encouraged’ to increase their total sleep duration, with little consideration of how this might be achieved. In the future, it will be important to determine whether, in athletes who obtain sufficient sleep at night (i.e., 8 h), daytime napping affects the quantity and/or quality of the ensuing nighttime sleep.

Athletes often experience situations that prevent them from spending adequate time in bed at night to obtain sufficient sleep. The demands of training, competition and travel can each result in delayed bedtimes or earlier rise times than usual, such that time in bed is substantially reduced [1,2,3,4,27,28,29]. In such situations it may be possible to offset the impact of these demands on nighttime sleep duration by incorporating a daytime nap into the schedule. For example, in situations where early morning training sessions are a regular occurrence, a nap in the early afternoon would be a reasonable strategy to ensure adequate sleep is obtained in that 24 h period when sleep duration may otherwise be truncated [4]. Similarly, morning or afternoon naps could be used on days when athletes must compete in the evening to reduce the impact of delayed bedtimes on subsequent sleep duration [2]. In either of these situations, however, one potential issue to consider is that the immediate benefit of napping may be reduced by sleep inertia. Sleep inertia is a term used to describe the reduced vigilance and impaired performance during the period that follows upon awakening [35]. However, there is very good evidence to indicate that athletes’ response times and sprint ability are not affected by sleep inertia following a 1 h or 2 h daytime nap provided an adequate warm-up undertaken after waking [36].

There are some delimitations that should be considered when interpreting the results of this study. The study was not designed to examine the composition of daytime naps, but rather, to determine whether ‘splitting’ sleep between a nighttime sleep episode and a daytime nap affected overall sleep duration and/or physical performance after waking from a nap [24]. Ideally, athletes should have been provided with the same duration of time in bed on the night prior to each nap. It is possible that the difference in homeostatic sleep pressure immediately prior to the 1 h and 2 h naps could account for some of the observed differences in sleep architecture between the naps. From a practical perspective however, the amount of sleep obtained on the night prior to the 1 h (7.3 h) and 2 h (6.3 h) naps is within the range of usual sleep duration reported for elite athletes (i.e., ~6.0–7.5 h) [1,4,37,38,39]. It seems reasonable then to assume that the composition of sleep in the daytime naps observed in the present study does reflect what an athlete might typically experience if they did attempt to nap during the day having spent less than 9 h in bed the night before a nap. Furthermore, the experimental protocol was conducted over three consecutive nights and days without a washout period between conditions. The lack of washout period may have resulted in a ‘carry-over’ of sleep pressure between conditions. However, participants completed the nap conditions in a counterbalanced order. This ensured that any carry-over effects were spread evenly across conditions. It is also important to note that the start time of each nap (14:00 and 15:00) coincides with the secondary peak in sleep propensity that usually occurs in the mid-afternoon in ordinarily diurnally active individuals [24]. Naps that are initiated earlier or later in the day may not result in the same sleep onset latency or the same composition of sleep reported in this study. Finally, participants were provided with ideal sleeping conditions (i.e., cool, dark, sound-attenuated bedrooms) and an untrained control group was not included in the experimental design. It is possible that the composition of daytime naps observed in the present study simply reflects the responses of healthy young males to sleeping under ideal conditions and is independent of the training status of the participants. The use of a small group of healthy young male athletes may also mean the results of the present study are not generalizable to female athletes or older athletes since daytime sleepiness and sleep propensity are affected by sex and age [40,41].

## 4. Materials and Methods

Data were collected as part of a study that was designed to examine the impact of different sleep strategies on performance in athletes [24]. A repeated measures cross-over design was employed in which participants completed three conditions in a counterbalanced order over three consecutive nights/days. The conditions were: a 9 h sleep opportunity from 22:00 to 07:00; an 8 h sleep opportunity from 23:00 to 07:00 with a 1 h nap opportunity the following day from 15:00 to 16:00; and a 7 h sleep opportunity from 00:00 to 07:00 with a 2 h nap opportunity the following day from 14:00 to 16:00. The data from the two conditions involving daytime naps are reported in the present study.

Twelve well-trained, semi-professional soccer players (age: 18.3 ± 1.0 years; mean ± SD) gave their written informed consent to participate in the study as volunteers. The participants usually trained five times per week and played one game on the weekend. The participants completed field-based training sessions three times a week (90 min/session) and completed gym-based training sessions twice a week (45 min/session). Participants were excluded from the study if they reported a clinical diagnosis of a sleep disorder. None of the participants were taking medication or supplements to assist with sleep. The study was approved by Central Queensland University’s Human Research Ethics Committee and was conducted in accordance with The Code of Ethics of the World Medical Association (Declaration of Helsinki). The study was funded by the Australian Institute of Sport (AIS) High Performance Sport Research Fund. The AIS did not participate in data collection, nor were they involved in the analysis and interpretation of the data.

Sleep was recorded using polysomnography (Grael; Compumedics, Melbourne, Australia) with a standard montage of electrodes (three electroencephalograms, two electrooculograms and a submental electromyogram). Sleep records were manually scored in 30 s epochs by a single technician according to established criteria [42]. The following variables were calculated: sleep onset latency, total sleep time, time spent in stage N1, stage N2, stage N3, and rapid eye movement (REM) sleep, wake after sleep onset, stage N3 onset latency, REM onset latency, sleep efficiency (total sleep time divided by time in bed × 100), arousals during sleep, awakenings, and stage shifts. Subjective sleep quality was assessed using a 7-point scale, where 1 = ‘extremely poor’ and 7 = ‘extremely good’.

The study was conducted in a windowless and sound-attenuated sleep laboratory. Apart from differences in the duration of the daytime nap opportunity between the two conditions, the protocol was identical each day. Participants consumed breakfast between 07:10 and 07:50 h, after which they attended a training session from 08:30 to 11:30. Participants were driven as a group to/from training each day by a member of the research team. The training session consisted of a combination of on-field skills and strength and conditioning tasks and was identical on both days. Upon returning to the sleep laboratory, participants consumed lunch between 12:00 and 12:30. Participants then had free time to read or watch television. In the 30 min prior to each nap, electrodes were attached to participants. In the 2 h nap condition, participants were in bed between 14:00 and 16:00 and in the 1 h nap condition, participants were in bed between 15:00 and 16:00. Participants were instructed to close their eyes and attempt to sleep and remained in bed lying down for the duration of the nap. The lights were extinguished in both nap conditions (<0.03 lux). At the end of each nap at 16:00, the lights were switched on (~350 lux; 6-foot angle of gaze) and the electrodes were removed. The target temperature for the laboratory was 21–23 °C.

Differences in sleep variables between the 1 h and 2 h naps were examined using generalized estimating equations (GEE) models [43,44]. GEE models can account for within-subject correlation inherent in repeated-measures, cross-over designs while remaining robust against violations of normality in the dependent variables [45,46,47]. A separate GEE model was constructed for each dependent variable. Each model utilized a linear response structure paired with the Huber–White sandwich estimator [48]. The within-subject dependency across the two nap conditions was modelled using an exchangeable working covariance matrix structure. In all models, the categorical variable ‘nap condition’ (1 h vs. 2 h) was entered as a fixed factor, and the continuous variable ‘prior total sleep time’ (i.e., total minutes of sleep obtained during the nighttime sleep opportunity immediately preceding each nap) was entered as a covariate. In each model, the 2 h nap condition served as the reference group. Main effects were evaluated using Wald chi-square (*χ*^2^), and unstandardized regression coefficients (*β*) were reported to indicate the direction and magnitude of effects. Cohen’s *d* equivalent effect sizes were calculated [26] and interpreted as small (0.2), medium (0.5), and large (0.8) [49].

Differences in sleep variables between the 7 h and 8 h nighttime sleeps preceding each nap were examined using paired *t*-tests for normally distributed data and Wilcoxon signed-rank tests for non-normally distributed data. The probability distribution of sleep stages during each nap condition was examined using a sleep histogram. Sleep stage probability was calculated in 1 min intervals as the percentage of epochs across all participants that were scored as either Stage N1, Stage N2, Stage N3, REM sleep or Wake. Results were considered significant at *p* < 0.05. Statistical analyses were performed using IBM SPSS Statistics (Version: 29.0.0.0, IBM, Armonk, NY, USA).

## 5. Conclusions

In the present study, healthy young male semi-professional soccer players were able to initiate sleep easily during a daytime nap, despite spending 7–8 h in bed on the night prior to the nap. In the 1 h nap, participants cycled from light sleep through to deep sleep; in the 2 h nap, participants cycled from light sleep through to deep sleep and REM sleep. Daytime napping may be a suitable strategy for athletes who wish to supplement inadequate nighttime sleep or increase total sleep duration beyond the usual recommendation.

## Figures and Tables

**Table 1:** Sleep variables during the 1 h and 2 h daytime naps.

	1 h Nap		2 h Nap	
Variable	Mean (SD)	Median (IQR)	Mean (SD)	Median (IQR)
Sleep onset latency (min)	6.5 (10.9) *	3.2 (4.2)	7.5 (10.1) *	4.5 (5.6)
Total sleep time (min)	52.4 (10.7) *	56.0 (4.0)	105.0 (15.3) *	109.2 (11.7)
Stage N1 (min)	4.2 (2.5)	4.0 (4.9)	9.0 (5.5)	8.0 (9.4)
Stage N2 (min)	15.7 (6.1)	15.5 (7.4)	46.4 (14.0)	51.5 (22.5)
Stage N3 (min)	30.4 (12.6)	32.7 (24.1)	36.2 (8.6)	37.2 (11.4)
Stage REM (min)	2.1 (7.2) *	0.0 (0.0)	13.4 (15.7)	7.5 (21.0)
WASO (min)	1.0 (1.4) *	0.7 (1.4)	7.4 (12.6) *	3.0 (5.4)
Stage N3 latency (min)	15.2 (7.2) *	13.7 (5.7)	18.2 (7.9)	16.0 (11.1)
Stage REM latency (min)	55.4 (17.0) *	60.0 (0.0)	80.7 (33.4)	82.2 (51.1)
Sleep efficiency (%)	87.4 (17.9) *	93.3 (7.3)	87.5 (12.7) *	91.0 (9.8)
Stage N1 (%)	8.1 (4.5)	7.4 (7.6)	8.9 (6.1)	6.9 (8.0)
Stage N2 (%)	31.4 (13.1)	30.0 (24.4)	43.8 (10.8)	45.3 (17.5)
Stage N3 (%)	56.7 (18.4)	58.9 (31.7)	35.3 (9.9)	37.3 (15.1)
Stage REM (%)	3.6 (12.7) *	0.0 (0.0)	12.0 (13.2)	8.2 (18.0)
Arousals (count/hour)	5.8 (3.3)	5.2 (5.2)	10.0 (3.4)	9.7 (4.8)
Stage shifts (count)	16.1 (8.3)	16.5 (15.2)	36.8 (9.3)	38.5 (11.7)
Awakenings (count)	1.6 (1.9) *	1.5 (2.0)	5.0 (2.3) *	5.0 (2.2)
Sleep quality (units)	4.7 (1.1) *	5.0 (0.7)	5.2 (0.9)	5.0 (1.7)

Abbreviations: REM, rapid eye movement sleep; WASO, wake after sleep onset; SD, standard deviation; IQR, interquartile range. * indicates variables that are not normally distributed according to the Kolmogorov–Smirnov test for normality.

**Table 2:** Generalized estimating equation models comparing sleep variables between the 1 h and 2 h daytime naps.

	Adjusted Mean (SE)		Parameter Estimates and Effect Size					
Variable	1 h Nap	2 h Nap	*β*	SE	95% CI for *β*	Wald *χ*^2^	*p* Value	*d*
Sleep onset latency (min) *	6.0 (2.8)	8.1 (3.2)	−2.1	0.5	−3.2 to −1.1	15.64	<0.001	−0.2
Total sleep time (min) *	42.9 (2.8)	114.5 (3.0)	−71.5	5.2	−81.7 to −61.3	188.45	<0.001	−2.4
Stage N1 (min)	3.7 (1.0)	9.5 (1.7)	−5.7	1.9	−9.5 to −2.0	9.12	0.003	−1.2
Stage N2 (min) *	12.2 (1.8)	49.9 (3.6)	−37.6	3.7	−45.0 to −30.3	100.59	<0.001	−2.0
Stage N3 (min)	29.2 (3.2)	37.4 (3.3)	−8.2	5.1	−18.3 to 1.8	2.56	0.110	−0.7
Stage REM (min *)	0.0 (0.0) ^†^	16.0 (4.9)	−16.4	5.7	−27.7 to −5.3	8.32	0.004	−1.2
WASO (min)	2.1 (1.8)	6.3 (2.7)	−4.2	3.0	−10.2 to 1.8	1.90	0.168	−0.4
Stage N3 latency (min)	14.0 (1.9)	19.3 (2.5)	−5.3	2.6	−10.3 to −0.2	4.13	0.042	−0.7
Stage REM latency (min) *	62.1 (4.1)	74.1 (10.5)	−12.0	10.1	−31.9 to 7.9	1.40	0.237	−0.4
Sleep efficiency (%) *	75.9 (5.2)	99.1 (4.2)	−23.2	9.1	−41.0 to −5.4	6.53	0.011	−1.5
Stage N1 (%)	8.3 (1.6)	8.7 (1.8)	−0.4	2.3	−4.9 to 4.1	0.03	0.858	−0.1
Stage N2 (%)	29.5 (4.3)	45.8 (3.3)	−16.3	5.3	−26.6 to −5.9	9.43	0.002	−1.2
Stage N3 (%)	59.7 (4.7)	32.4 (3.2)	27.2	5.4	16.6 to 37.8	25.40	<0.001	1.5
Stage REM (%) *	1.1 (2.8)	14.5 (4.3)	−13.3	5.1	−23.4 to −3.3	6.75	0.009	−1.0
Arousals (count/hour)	5.5 (0.8)	10.3 (1.3)	−4.8	1.6	−7.9 to −1.6	8.60	0.003	−0.3
Stage shifts (count) *	14.9 (2.1)	38.0 (2.8)	−23.1	2.3	−27.6 to −18.6	100.00	<0.001	−1.7
Awakenings (count)	1.8 (0.5)	4.7 (0.7)	−2.9	0.5	−4.0 to −1.9	30.32	<0.001	−1.1
Sleep quality (units) *	4.9 (0.3)	5.0 (0.3)	−0.1	0.3	−0.6 to 0.5	0.03	0.858	−0.1

Abbreviations: REM, rapid eye movement sleep; WASO, wake after sleep onset; SE, robust standard error; *β*, unstandardized regression coefficient; CI, confidence interval; Wald *χ*^2^, Wald chi-square statistic for the main effect of nap condition (*df* = 1); *d*, Cohen’s *d* equivalent effect size calculated using the Feingold Method [26]. Adjusted means and standard errors are estimated marginal means derived from GEE models. ^†^ Adjusted mean mathematically converged to a negative value (−0.5) due to zero-bounding and truncated to 0.0 for physical interpretation. All models are adjusted for the covariate of total sleep time prior to the nap (mean total sleep time prior to 1 h and 2 h nap = 409.5 min). * indicates the main effect of total sleep time prior to the nap (i.e., covariate) is statistically significant for this dependent variable (*p* < 0.05). The 2 h nap condition serves as the reference group (*β* = 0).

## Data Availability

The original contributions presented in this study are included in the article. Further inquiries can be directed to the corresponding author.

## Abbreviations

The following abbreviations are used in this manuscript:

CI	Confidence interval
GEE	Generalized estimating equations
IQR	Interquartile range
REM	Rapid eye movement
SD	Standard deviation
SE	Robust standard error
WASO	Wake after sleep onset
